# Efforts at COVID-19 Vaccine Development: Challenges and Successes

**DOI:** 10.3390/vaccines8040739

**Published:** 2020-12-06

**Authors:** Azizul Haque, Anudeep B. Pant

**Affiliations:** 1 One Medical Center Drive, Department of Microbiology and Immunology, Geisel School of Medicine at Dartmouth, Lebanon, NH 03756, USA; 2 New Orleans East Hospital, 5620 Read Blvd, New Orleans, LA 70127, USA; Anudeep.Pant@lcmchealth.org

**Keywords:** pandemics, severe acute respiratory syndrome coronavirus 2 (SARS-CoV-2), COVID-19 disease, vaccines, trials, public health

## Abstract

The rapid spread of SARS-CoV-2, the new coronavirus (CoV), throughout the globe poses a daunting public health emergency. Different preventive efforts have been undertaken in response to this global health predicament; amongst them, vaccine development is at the forefront. Several sophisticated designs have been applied to create a vaccine against SARS-CoV-2, and 44 candidates have already entered clinical trials. At present, it is unclear which ones will meet the objectives of efficiency and safety, though several vaccines are gearing up to obtain emergency approval in the U.S. and Europe. This manuscript discusses the advantages and disadvantages of various vaccine platforms and evaluates the safety and efficacy of vaccines in advance stages. Once a vaccine is developed, the next challenge will be acquisition, deployment, and uptake. The present manuscript describes these challenges in detail and proposes solutions to the vast array of translational challenges. It is evident from the epidemiology of SARS-CoV-2 that the virus will remain a threat to everybody as long as the virus is still circulating in a few. We need affordable vaccines that are produced in sufficient quantity for use in every corner of the world.

## 1. Background

The continued unabated spread of the SARS-CoV-2 virus that causes COVID-19 disease poses a significant threat to human health. This condition is driven by the difficulty in containing the virus and by the non-availability of vaccines and therapeutics. Here, we review the strategies applied and the progress made in the development of efficient and safe vaccines. It is vitally important to strategize the procurement and introduction of vaccines once they are created and medically approved. We project solutions to overcome some of the translational hurdles and propose strategies for the global deployment of vaccines. We searched PubMed, Web of Science, Google Scholar, WHO, and CDC websites, as well as vaccine manufacturers’ websites, for updated developments to formulate this comprehensive review.

## 2. Introduction

The pandemic caused by the newly discovered SARS-CoV-2 virus has propagated sociological, psychological, and economic crises around the globe. Extensive measures such as widespread testing and strict isolation of infected individuals is necessary to impede further spreading; however, implementing these containment procedures poses a significant challenge. The virus is responsible for more than 64 million confirmed cases globally with more than 1.487 million deaths as of 2 December 2020 [1]. The continued spread of this coronavirus (CoV) highlights the importance of global efforts in developing vaccines and therapeutics. A COVID-19 vaccine that provides some degree of immune protection could allow lockdowns and social distancing to be relaxed. There are no vaccines proven yet to protect humans against the virus. Around 44 different coronavirus vaccines are currently under clinical trials in order to determine their efficacy and safety profile [2].

This paper is distinct from other published review articles on COVID-19 vaccines through its comparison of the strengths and weaknesses of vaccine candidates developed using different technological approaches, the efficacy and safety of the various candidate vaccines, and anticipated challenges beyond vaccine development, such as vaccine acceptance, acquisition, and global deployment.

## 3. SARS-CoV-2 Biology

SARS-CoV-2 is a positive-sense, single-stranded RNA virus and is genetically close to other coronavirus (CoV) strains such as SARS-CoV and MERS-CoV [3]. Bats serve as a natural reservoir for all three of these viruses [3]. Infection of humans likely occurred through intermediate hosts, such as pangolins in the case of SARS-CoV-2 [4]. Being RNA viruses, CoVs readily evolve by mutation and homologous and non-homologous recombination, which expand their host range.

**Figure 1:**
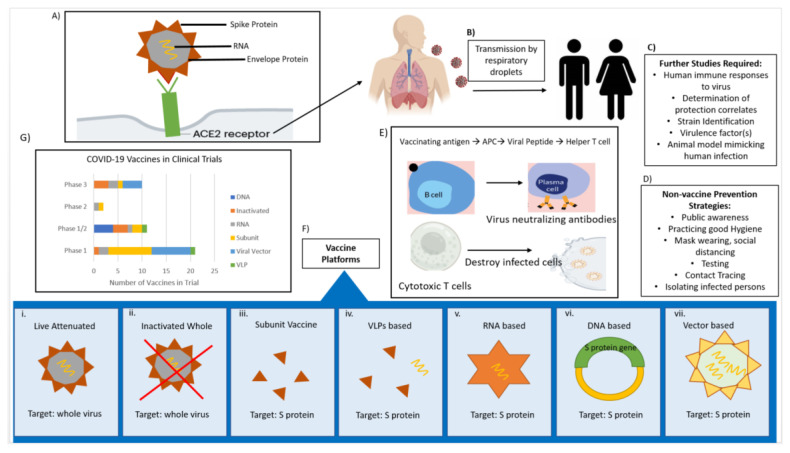
Portraying the biology of severe acute respiratory syndrome coronavirus 2 (SARS-CoV-2), mode of virus transmission, non-vaccine prevention strategies, and potential vaccine candidates that have progressed to clinical trials. (**A**) Viral structure and infection by binding of S protein to host receptor ACE2, (**B**) the transmission pathways, (**C**) identification of areas for further studies facilitating vaccine development, (**D**) non-vaccine strategies to contain the spread of infection, (**E**) potential vaccine may act through the production of neutralizing antibodies and/or cytotoxic CD8+ T cells that destroy the infected cells, (**F**) vaccine platforms for creating anti-SARS-CoV-2 vaccines, (**G**) number of potential vaccines entered into clinical trials.

CoVs are spherical in shape with characteristic club-like projections on the surface, which are referred to as “spikes”. The virus membrane contains four structural components, namely the spike (S), envelope (E), membrane (M), and nucleocapsid (N) proteins (Figure 1). The (S) protein facilitates binding to the trans-membrane angiotensin converting enzyme (ACE) 2 host receptor in SARS-CoV-2; (S) protein is the primary determinant for host transmissibility and pathogenicity [5,6,7] and is the main target for neutralizing antibodies, and therefore is considered as a primary target for vaccine design [8].

## 4. SARS-CoV-2 Transmission and Disease

Several coronaviruses like SARS-CoV, NL63, and SARS-CoV-2 use ACE2 as the host receptor [9]. ACE2 is highly conserved among mammals, thus facilitating interspecies transfer [10]. SARS-CoV-2 infects airway epithelial cells through interactions with the trans-membrane ACE2. Tissue monocytes/macrophages express ACE2 to a significantly lower extent. Analysis of the receptor binding motif (RBM) in the spike protein showed that most of the amino acid residues essential for receptor binding were conserved between SARS-CoV and SARS-CoV-2, suggesting that the 2 CoV strains use the same host receptor for cell entry [11]. The virus spike protein is a trimeric multidomain glycoprotein that is able to specifically recognize ACE2 via its receptor binding domain (RBD). The conformational changes induced by the RBD/ACE2 interactions ultimately result in viral and cellular lipid membrane fusion and hence in cell infection. The RBD and ACE2 represent ideal targets to counteract the SARS-CoV-2 infection [12]. Hoffmann et al. investigated if SARS-CoV-2 entry is also dependent on spike protein priming by TMPRSS2 [13]. Specifically, spike protein priming by the serine protease TMPRSS2 is crucial for SARS-CoV infection of target cells and spread throughout the host. Researchers found that the binding affinity of the S protein of SARS-CoV-2 to its target ACE2 on human cells is markedly higher than the spike from the SARS virus from 2002 [14].

Importation of this new coronavirus to a naïve population may explain the profuse seeding of COVID-19 at the “epicenters”, where spatial closeness facilitates rampant transmission. Viral titers are highest in the earliest phases of infection [15]. The ability for asymptomatic individuals to still shed the virus provides a likely explanation for the high infection rate [16,17]. A key factor in the transmissibility of COVID-19 is the high level of SARS-CoV-2 shedding in the upper respiratory tract compared to SARS-CoV-1, where replication occurs mainly in the lower respiratory tract [18]. Recent studies suggest that the newly evolved G614 strain is 3–9 times more infective than the old strain D614 [19,20].

SARS-CoV-2 is transmitted primarily though droplets, i.e., respiratory secretions and saliva. There are reports of transmission in the absence of clear symptoms [21,22]. Recently, the possibility of short-range aerosol spreading of virus has been emphasized [23,24]. However, the larger droplets from coughs and sneezes are still the primary source of infection [25]. The risk of infection increases the longer and closer a person is to someone who has the virus, and poorly ventilated enclosed crowded spaces are riskier than the outdoors. Some studies have suggested a link between the amount of virus exposure and the severity of illness [26,27], though the evidence of this remains inconclusive, as the severity of disease is more likely to be dependent on the individual’s immune system.

Many patients that contracted SARS-CoV-2 and developed severe disease have comorbidities like diabetes, cardiovascular disease, and immunocompromised conditions [28,29,30]. Older people are likely to have damaged lungs due to smoking, inhaling polluted air particles, and weakened immunity, making them more susceptible to infection. Younger adults are also being hospitalized in the U.S. and elsewhere. Infected children appear to be less symptomatic and may act as carriers of viral infection [31]. 

## 5. The Host’s Immune Responses to SARS-CoV-2

A recent study demonstrated that antibodies generated in asymptomatic individuals tend to fade away in two to three months [32,33]. Antibodies are a critical component of immunity—especially the ones that “neutralize” the virus. A good vaccine will try to replicate that kind of natural protection.

Measuring both humoral and cellular immunity to SARS-CoV-2 is vital for vaccine development. Grifoni et al. provided critical knowledge that showed circulating SARS-CoV-2-specific CD8+ and CD4+ T cells were identified in ~70 and 100% of COVID-19 patients, respectively. CD4+ T cell responses to the S protein were robust and correlated with the magnitude of the anti-SARS-CoV-2 IgG and IgA titers. Interestingly, T cell reactivity to SARS-CoV-2 pooled epitopes was also detected in non-exposed individuals, indicating possible cross-reactivity with other CoVs that are commonly circulating in the population [34]. In another study, memory T cells were found in individuals who recovered from SARS-CoV-2 infection [35]. Ni et al. found a correlation between neutralizing antibody titers and the numbers of virus-specific T cells in convalescent subjects [36]. Interestingly, robust SARS-CoV-2-specific CD4+ and CD8+ responses are seen in convalescent individuals with asymptomatic or mild COVID-19 [37], who are showing positive serostatus. Whether such a response could prevent recurrent episodes of severe COVID-19 is yet to be proven. In a recent study, robust T cell responses to SARS-CoV-2 to spike protein and as well as to nucleoprotein and membrane proteins have been shown to persist at least for six months after even mild or asymptomatic infection [38]. This provides evidence that T cell immunity to SARS-CoV-2 may last longer than antibody immunity. However, the critical question remains whether these persistent T cells provide efficient protection against re-infection. Interestingly, T cell reactivity to SARS-CoV-2 has been described in non-exposed individuals indicating possible cross-reactivity with other CoVs that are commonly circulating in the human population [39]. Other studies reported generation of putative cross-reactive responses between SARS-CoV-2 proteins and pneumococcal or diphtheria toxoid (DT) antigens in individuals that previously received pneumococcal or DTP vaccines [40,41]. The role of this cross-reactivity in protection or immunopathology has not yet been defined.

Immune responses, such as marked inflammatory responses, develop after infection with SARS-CoV-2 [42]. This condition is associated with significant synthesis of various proinflammatory cytokines and chemokines [43,44]. These observations are consistent with the possible induction of innate immune responsiveness in infected persons. A recent study by Carsetti et al. demonstrated that a high frequency of natural killer cells and early increases in IgA, IgM, and IgG are associated with asymptomatic SARS-CoV-2 infection, while high levels of monocyte induction and persistent levels of IgA and IgG produced in late stages of infection characterize severe disease. This suggests that only severe COVID-10 disease may result in protective memory established by an adaptive immune response [45].

Recent studies demonstrate the induction of cytokine storms by SARS-CoV-2 [46], and that persistent and high pro-inflammatory responses that progress to cytokine hyperactivation (cytokine storm) appear to cause acute respiratory distress syndrome (ARDS) and further organ injury in a subset of COVID-19 patients [47]. We previously postulated a similar role of cytokine storms in advanced pneumonia in H5N1 influenza and proposed development of therapeutic vaccines directed at diminishing the cytokine storm [48].

Severe COVID-19 disease pathogenesis is marked by dysregulation in innate immune cells, and recent studies suggest that some of the immune responder cells induced by SARS-CoV-2 infection may be partially activated or altogether dysfunctional [49]. Furthermore, recent literature suggests that CoV can manipulate interferon (IFN) responses; effective innate immune responses against viral infection are heavily dependent on IFN responses (and the downstream pathways) that control viral replication as well as the tapping of the adaptive arm of the immune system. Of note, structural proteins of both SARS-CoV and MERS-CoV have been shown to act as IFN antagonists [50].

Scientists are still grappling with the issue of coronavirus and immunity. Never in the history of public health has herd immunity been recommended as a strategy for responding to an outbreak, let alone in a pandemic against a novel respiratory virus. Herd immunity is a flawed goal without a vaccine, as many infectious conditions never reach that point, e.g., measles, malaria, AIDS, and flu. If the evidence of increasing reinfection is confirmed, it is unlikely that we would ever reach herd immunity against SARS-CoV-2.

Researchers have been looking to understand how immunity to the new virus might work and how to design a vaccine. The role of T or B cells in asymptomatic, symptomatic, cured, or re-infected individuals has not been thoroughly investigated. We do not know if repeated infections are required to develop sustained immunity. Further studies into the immune evasion mechanisms of SARS-CoV-2 could be vital for efficacious vaccine development. While the role of the innate immune response in memory immunity is not well understood, further studies into the innate immune response would help design effective vaccines that target and hamper viral components that dysregulate the innate immune response or aid in immune evasion mechanisms. Further investigation into to these unknown aspects of host immunity will explain mechanisms of protection and aid in defining protection correlates, which are essential to validate any vaccine’s utility in a pandemic. A good vaccine response is characterized by the robust induction of humoral and cell-mediated immunity, as well as the stimulation of the innate immune system (using adjuvants or other mechanisms) to further drive the adaptive response.

## 6. Vaccine Development Platforms for SARS-CoV-2

Currently, there are no licensed vaccines to prevent SARS-CoV-2 infection, though research is happening at breakneck speed, and significant progress has been made toward this goal. About 240 vaccines are in early development, with 44 in clinical trials and nine already in the final stage of testing on thousands of people (Figure 1). Vaccines normally take years to develop, yet researchers hope to achieve the same goal in only a few months. Most experts think a vaccine is likely to become widely available by mid-2021, about 12–18 months after the new virus first emerged. It is worth noting that four coronaviruses already commonly circulate in human beings. They cause common cold symptoms, and we do not have vaccines for any of them. The goal should be to produce a safe and highly effective COVID-19 vaccine; vaccination with just 70 or 75 percent efficacy can dampen the outbreak. For example, the flu vaccine is not 100 percent effective but is still beneficial.

Vaccines against SARS-CoV-2 are likely to target the viral S protein, due to its vital role in viral infectivity. Other viral proteins such as the N protein, E protein, and non-structural protein 16 (NSP16) could also be considered as targets for vaccine development. In view of the lower mutation rates and relatively conserved sequences of the coronavirus N gene, it could be another useful target for vaccines and diagnostics. Epitope characterization of the structural protein NP-specific T cells showed recognition of protein fragments with low homology to common cold human coronaviruses but conserved amongst betacoranaviruses [51]. The best-case scenario would be the development of a universal vaccine that can be given only once. Such a vaccine would provide protection against all, or at least most, of the many strains of SARS-CoV-2, including future pandemic coronaviruses.

There is an urgent need to develop animal models that mimic the natural infection in humans. Vaccines need to be tested in animal models for their safety and efficacy. Some of the candidate vaccines have been tested in monkeys; however, they do not develop the severe symptoms that SARS-CoV-2 causes in humans.

The vaccine platforms used to develop anti-SARS-CoV-2 include live attenuated vaccines, inactivated whole-virus vaccines, subunit vaccines, virus like particle (VLP) vaccines, mRNA-based vaccines, DNA-based vaccines, and viral vector-based vaccines [52]. Each method has different strengths and weaknesses, which are summarized in Table 1.

The main method of vaccination for decades has been to use the original virus, attenuated by various methods, as it often produces long-lasting immunity. The measles, mumps, and rubella (MMR) vaccine is made by using weakened viruses that cannot cause a full-blown infection. Some scientists, particularly those in China, are using this approach.

An alternative method to using the whole virus is to pick a specific protein, protein fragment, or subunit of a pathogen to create a vaccine. Vaccines can also be developed by using VLPs, which are a sort of intermediate between protein subunits and inactivated or attenuated viruses. Researchers at the University of Queensland, in Australia, have developed a “molecular clamp” technique, which helps the viral proteins retain their shape, enabling the immune system to mount a much stronger immune response.

Another alternative vaccine platform involves the use of nucleic acids. For mRNA-based vaccines, a synthetic version of the mRNA that a virus uses to build its infectious proteins is delivered into the human body, whose cells read it as instructions to build that virus’ proteins. The immune system detects those proteins and induces an immune response against the virus. DNA vaccine platforms use a circular DNA plasmid, coding for some antigen protein, engineered with strong promoter signals and stop signals at both ends of the sequence. There is no licensed human vaccine that uses nucleic acids.

Viral vector-based vaccines use virus like adenovirus or vaccinia virus to carry DNA into human cells. The DNA contained in the virus encodes antigens that, once expressed in the infected human cells, elicit an immune response.

Both DNA and mRNA methods have several potential advantages over traditional vaccines, but a significant one is the speed at which a candidate vaccine can be created [53,54]. Having known the genetic sequence of the new coronavirus, it was possible to develop a vaccine within a few weeks. There is another approach that is sometimes called “plug-and-play”; DNA or RNA from a large number of germs can be “plugged” into the same vaccine platform [55].

## 7. Vaccines in Advanced Stage

The vaccines that are considered to be front-runners include the following: Sino Biotech’s CoronaVac, which is an inactivated virus vaccine; Moderna’s mRNA1273, which is an mRNA candidate; Johnson & Johnson’s JNJ-78436735, which is an adenovirus-based vaccine; Pfizer’s BNT162b2, which is an mRNA-based vaccine; the University of Oxford’s candidate ChAdOx1 nCoV-19, which is an adenovirus-based vaccine; Sinovac’s SARS-CoV-2 vaccine, which is an inactivated candidate; CanSino’s Ad5-nCoV, which is a viral vector vaccine; Russian Gamaleya Institute’s Sputnik V, which is an adenovirus-based vaccine; and Inovio’s INO4800, which is a DNA plasmid vaccine.

CoronaVac is an inactivated vaccine developed by Sinovac Biotech. Preliminary results demonstrated that the vaccine generated antibodies capable of neutralizing 10 strains of SARS-CoV-2 [56]. Results from Sinovac’s phase two human trials also seem promising; the biotech company has released preprint results that showed the vaccine produced neutralizing antibodies with no severe adverse reactions. They are currently gearing up to conduct phase three trials in Brazil, Indonesia, and Bangladesh.

Recent preliminary data from Pfizer and BioNTech suggest that the vaccine is 95% effective based on 170 cases of COVID-19 developing in volunteers. Of note, eight individuals were in the group given the vaccine. The companies Pfizer and BioNTech said that the vaccine’s efficacy was consistent across age, race, and ethnicity. The most common serious adverse event was fatigue, with 3.7 percent of volunteers reporting tiredness after they took the second dose. Two percent of volunteers reported a headache after the second dose. Older adults reported fewer and milder side effects, the companies said. Although the full trial data are not yet published, both companies have stated their intent to apply for authorization for emergency use of the vaccines in the U.S.

Moderna’s mRNA vaccine candidate injects coronavirus’ genetic information into a non-virulent viral vector, which then creates viral proteins that mimic the coronavirus. To date, mRNA vaccines have not been licensed for use, though preliminary results of Moderna’s trials show promise. If approved, both the mRNA-based vaccines made by Moderna and by Pfizer and BioNTech would be the first of its kind. Vaccination of nonhuman primates with the vaccine induced antibody levels exceeding those in human convalescent-phase serum and produced type 1 helper T-cell (Th1)–biased CD4 T-cell responses and low to no Th2 or CD8 T-cell responses [57].

Recent studies demonstrate that the mRNA vaccine manufactured by Moderna can stimulate the production of antibodies, which are able to neutralize the virus in laboratory samples [57,58]. It would be a big breakthrough if the same effects could be replicated in larger human trials and demonstrate that the immune response induced by the vaccine is strong enough to protect humans from developing COVID-19 pathogenesis. This vaccine requires two doses, four weeks apart. There remain concerns regarding the severe side effects experienced by a small number of participants who were given the highest dose of the vaccine. While phase two results are still being monitored, Moderna announced that it started phase three trials in 30,000 U.S. participants. Interim trial results released by Moderna suggest that their vaccine induced similar protection levels as the vaccine produced by Pfizer and BionTech. The biotech company is poised to manufacture up to one billion doses a year since partnering with the Swiss manufacturer Lonza.

Similarly, a SARS-CoV-2 neutralizing antibody response was detected in both nonhuman primates and in humans when researchers tested another leading mRNA vaccine named BNT162b, which is manufactured by Pfizer in conjunction with German Biotech company BioNTech [59,60]. Like the Moderna vaccine, this one also produces T-cell responses specific to SARS-CoV-2 proteins and will soon start phase three trials to include 44,000 people across several countries. Pfizer is gearing up to provide 100 million doses through a contract with the U.S. government, which goes into effect when and if the drug is approved.

Researchers at Oxford University in conjunction with AstraZeneca, CanSino Biologics, and Johnson & Johnson all are using adenovirus vector-based vaccine platforms to make COVID-19 vaccines. In this technology, research teams have transferred the SARS-CoV-2 spike protein into a weakened version of an adenovirus. When this adenovirus is injected into humans, the hope is that the spike protein will trigger an immune response.

CanSino Biologics is using the Ad-5 vector, whereas Johnson & Johnson is using the Ad-26 vector. The former vector infects populations in many parts of the world, and the later one is less prevalent; however, it is present in Sub-Saharan Africa and some parts of Asia. The adenovirus used in creating a COVID-19 vaccine by the Oxford lab is a nonhuman adenovirus vector obtained from feces of Chimpanzee, dubbed ChAdOx1. Preliminary results from the University of Oxford’s ChAdOx1 vaccine candidate’s clinical trial revealed that the vaccine could induce a potent antibody and T-cell response with only minor side effects such as headache and fatigue [61,62].

On 8 September, AstraZeneca paused clinical trials due to an adverse reaction in one of the trial participants. The details of the reaction remain unknown; however, following a thorough investigation by an independent party, the trials resumed in the U.K., Brazil, South Africa, and India but have remained on hold in the U.S. as of 23 September. AstraZeneca and Oxford have announced their plans to produce a billion doses of vaccine, which the manufacturers have said will be sold at cost.

Johnson & Johnson went on to develop multiple Ad26-based vaccines for viruses like HIV, Zika, and Ebola. The Ebola vaccine regimen uses a shot of an Ad26-based vaccine plus a booster shot of a different vaccine. That combination used by Johnson & Johnson makes it hard to draw comparisons to its COVID-19 vaccine, which uses only Ad26. A study published in *Nature* showed that the vaccine elicited neutralizing antibodies in monkeys and provided “complete or near-complete” protection with just one dose. The Johnson & Johnson trial is the biggest trial of the vaccine that we know of—60,000 adults from a variety of countries. The trial includes “significant representation” from older populations. As of 12 October 2020, Johnson & Johnson has temporarily paused all COVID-19 vaccine candidate clinical trials, including the Phase 3 ENSEMBLE trial, due to an unexplained illness in a study participant. While a few pauses are expected in trials involving large numbers of participants, concerns arise when serious adverse events are increasingly occurring. U.S. regulators have now given the green light to resume the trials of both the vaccines produced by AstraZeneca and Johnson & Johnson. Both these manufacturers have contracts to provide their vaccines to the U.S. government if approved by regulators.

CanSino has also developed a viral vector vaccine, which expresses and introduces the SARS-CoV-2 spike protein to the body. Preliminary reports published in *The Lancet* are promising and demonstrate the induction of a significant immune response after only one immunization [63,64]. No serious adverse effects have been documented. The Chinese government has approved the vaccine for military use only.

Petrovax, a Russian biopharmaceutical company, announced the start of phase three trials for their Ad5-nCoV vaccine on 15 August. The vaccine was made by the Gamaleya National Center and uses two strains of adenovirus 5. The vaccine is comprised of two injections; the second is given after 21 days to boost the immune response. Officials with the institute state that they have completed phases one and two studies and that they have seen robust antibody and cellular immune responses. After less than two months of testing, Russian officials have given the vaccine regulatory approval. Promising results from trials of the COVID-19 vaccine, Sputnik, reportedly show 92% protection; though unlike other researchers, this group has not released any data on safety or efficacy, which makes it difficult for independent assessment.

Adenovirus-based COVID-19 programs have garnered global attention for their scale and speed. Yet questions arise whether they can overcome their checkered past. Since adenoviral vectors are based on natural viruses that some of us might already have been exposed to, responses to the vaccines might be misdirected. Another concern is the dose of the vaccine; if the dose required to illicit an effective immune response is high, the result could be adenovirus-induced heightened inflammation, which would send the immune system into overdrive. To keep inflammation under control, the vaccines might have to be administered at low doses and with an adjuvant. Some vaccine experts opined that administering the vaccines as nasal sprays or pills may help in circumventing preexisting immunity to Ad5 in the bloodstream.

While most vaccine scientists agree that adenoviral vector vaccines are great at spurring T-cell immunity, we do not know how important that will be for preventing COVID-19 in humans. Most research has focused on the immune system’s antibody response to the virus. Adenoviral vector vaccines can induce antibody responses, but not as strong as those elicited by more traditional vaccines.

Inovio’s INO4800 is a DNA plasmid; two cellular candidates from Shenzhen Geno-Immune Medical Institute include LV-SMENP-DC, a dendritic cell vaccine that has been modified with lentivirus vectors to express viral proteins, and an artificial antigen-presenting cell (aAPC) vaccine along the same lines.

It has recently been reported that DNA vaccines are able to elicit measurable immune responses in immunized animals [65] and in humans in initial trials [66] that could lead to protection against SARS-CoV-2. Further studies are to be pursued to determine their efficacies and safety in larger human trials.

The standardization of endpoints is critical to effectively compare results across different studies. In the case of the current pandemic, endpoints of both nonclinical and clinical trials have already been defined by the FDA; however, it is up to the manufacturer whether to follow the FDA’s guidance or not. Currently, laboratory confirmed SARS-CoV-1, symptomatic COVID-19 infection, or a combination of the two are described as acceptable primary endpoints for vaccine efficacy trials [67]. There has been much discussion around whether or not the right endpoints are being studied [68], and if primary and secondary endpoints should differ by vaccine type [69]. Of note, the most important efficacy endpoint, protection against severe disease and death, is difficult to assess in phase 3 clinical trials [70].

Validity of a candidate vaccine should be assessed by an evaluation of humoral, cellular, and functional immune responses as appropriate to each of the included COVID-19 antigens. Measurement of cellular responses should be assessed by CD8+ and CD4+ T cell responses. The functional activity of immune responses should be tested in vitro in neutralization assays using either wild-type virus or pseudovirus. True efficacy could be determined via challenge infections; however, the ethics of this process have been questioned in the absence of a cure. Once this step has been carried out, the clinical signs need to be monitored for signs of protection or unintended toxic effects. Post-vaccination animal challenge studies and the determination of the type of nonclinical and clinical immune responses induced by the particular COVID-19 vaccine candidate can be used to ascertain the likelihood of the vaccine to induce vaccine-associated respiratory pathology in humans. A candidate vaccine against SARS-CoV-2 might act against infection, disease, or transmission, and a vaccine capable of reducing any of these elements could contribute to disease control [71]. A vaccine, whether fully or partially protective, would lead to lowering the “R” value (viral reproduction number), thus diminishing the viral spread.

Recent reports described that several COVID-19 vaccines in early trials stimulated a robust antibody response in vaccinated subjects. However, we do not know how much immunity they provide and for how long, and if they will protect humans against the virus in challenging infections. All the developers asserted that their vaccines elicited antibody levels like those seen in patients who have recovered from COVID-19. Of note, the antibody responses in convalescing patients varied widely, and that even matching those responses did not necessarily guarantee any degree of immunity.

Some developed countries, including the U.S.A. and U.K., have secured access to three other vaccines, namely Sanofi and GlaxoSmithKline’s protein adjuvant vaccine, Novavax’ protein adjuvant vaccine NVX-CoV2373, and Valneva’s inactivated virus vaccine VLA2001. These vaccines were developed by leveraging manufacturers’ already proven vaccine technologies. They are in the early stages of clinical trials, and preliminary data show promise; however, data on the efficiency and safety are yet to be published.

The immune responses elicited by sub-potent vaccines may exert selection pressure for the emergence of a mutated virus. Emergence of novel subtypes with antigenic changes could be a challenge for COVID-19 vaccine developers. It is thought that the SARS-CoV-2 virus has mutated relatively slowly so far—especially compared with other RNA viruses like HIV and influenza. However, a recent report has shown that mutations have made a new variant of SARS-CoV-2 virus, called G614, which has almost completely replaced the first version to spread in Europe and the U.S., which is called D614 [19]. Current vaccines being tested mostly target the spike protein, but they were made using older strains of the virus. It seems important to evaluate whether the newly mutated virus can be controlled by the vaccines that are under development.

## 8. Enhancing the Immune Response to a Vaccine

For some vaccines, a boosting protocol may be required to induce sufficient immunity, and appropriate vaccine adjuvants may be required to generate a sufficient immune response or for dose sparing. CpG 1018, MF59^®^, and AS03 are already approved for human vaccines, and their inclusion may expedite COVID-19 vaccine development process. One adjuvant, Protollin, induces both systemic and mucosal immune responses that could be evaluated in COVID-19 vaccines [72].

Small molecule toll-like receptor (TLR) 7/8 agonists have demonstrated potential as vaccine adjuvants, since they directly activate antigen-presenting cells (APCs) and can enhance both humoral and cellular immune responses, especially TH1 responses [73]. Other potential technologies might involve self-adjuvating recombinant or molecular vaccines that have built-in antigen-sparing properties.

Bacille Calmette Guérin (BCG), using different formulations of live bacteria, may have varying degrees of immune boosting ability. It is possible that BCG gives a bit of extra strength to the human immune system by efficiently bridging the innate immunity with the adaptive immunity encountering SARS-CoV-2. After abating multiple confounding factors, significant associations between BCG vaccination and reduced COVID-19 deaths were observed [74]. Countries with universal BCG vaccinations, such as Japan and South Korea, have infection and disease rates of COVID-19 as much as 100-fold lower than countries without universal vaccination policies, such as Italy, the Netherlands, and the United States. The elderly, who were vaccinated decades ago, may particularly benefit.

## 9. Translational Challenges

It is important to investigate whether vaccines confer full or partial protection, if this varies with the age, whether the vaccinated individuals are protected from recurrent infections, and, if immunized, whether people would need boosting (which would necessitate the manufacturing of billions of additional doses). Earlier animal experiments with vaccines against the related coronaviruses that cause SARS-1 and MERS demonstrated that low antibody levels could lead to aberrant immune responses. Some antibodies against the SARS-CoV-1 spike may mediate antibody-dependent enhancement (ADE) of infection [75]. There could also be cell-based enhancement, a category that includes allergic inflammation caused by Th2 immunopathology following vaccination [76].

Critical potency testing, standardization, and quality control of vaccines are required to determine the level of immune responses the vaccines generate. With the recent development of new technologies, vaccine manufacturing is not as difficult as proving that a vaccine is safe and effective. Another challenge is testing the vaccine, because this requires that people who volunteered for trials are infected. The experimental method of giving people the vaccine and then deliberately infecting them (challenge infection) could give quicker answers. The concern is that this process is too dangerous at this stage to infect individuals in the absence of a cure. Some vaccine developers like to adopt the Goldilocks approach for challenge infection. This would require enough virus to infect them, but not so much that it might overwhelm their immune system. Another option would be to give challenge infection with less virulent viruses if available. However, this may not mimic the natural infection process nor exclude the risk of disease following a challenge infection.

If a vaccine is developed, then there will be a limited supply, at least initially, so it will be important to prioritize recipient groups. Deciding on priority groups will be a difficult task in the case of COVID-19 because of the high rate of infection and mortality. Healthcare workers who encounter COVID-19 patients would top the list. The disease is most deadly in older people, so they would also be a priority if the vaccine is effective in this age group.

Production capacity could be a critical limitation to the deployment of pandemic vaccine globally. Some labs and pharmaceutical companies already are setting up plans to scale up production once medical authorities approve the vaccines. Prior discussion and fore planning are required to meet the huge logistical challenge of immunizing most of the world’s population. As in any pandemic, public and private sectors must mobilize to produce and distribute vaccines, if one is available, as quickly as possible.

Many of the vaccines being developed require two doses in order to be effective. For a world population of 7 billion, marked up-scaling of the manufacturing process is therefore required to produce double the vaccine doses. Not all vaccine technologies, especially those that are showing the most promising results in clinical trials against SARS-CoV-2, have been applied for such a largescale manufacturing before.

Some vaccines also require special devices to be used. There are some DNA-based vaccines in development that require an electroporation device for each dose. It uses a device the size of an electric toothbrush to generate a small electrical current that opens holes in the membrane of a cell, allowing a vaccine to enter. Although the device can be used multiple times, it is nonetheless an additional challenge in order to ensure there are a sufficient number of these.

There are questions around raw materials—both for the vaccine and glass vials—and refrigerator capacity. AstraZeneca has suggested their vaccine would need the regular cold chain between 2 °C and 8 °C. Some other candidate vaccines may need an ultra-cold chain—storage at −60 °C or colder before being diluted and distributed. This can be an immense task where infrastructure is weak and electricity supply and refrigeration is unstable. The WHO, UNICEF, and Médecins Sans Frontieres (MSF/Doctors Without Borders) already have effective vaccination programs in places around the world. Of note, developed countries have a strong cold-chain infrastructure, but many African countries and some Asian countries could face challenges in this regard. The procurement of vaccines and supply chain logistics will pose significant challenges due the unprecedented scale and nature of the virus.

Of note, the key to a successful immunization program is acceptance by the public. Without this critical piece, future public health responses would be crippled. Vaccine hesitancy sentiments pose a growing problem that have become embroiled in anti-mask, anti-lockdown ideologies. If cohorts of people refuse to have the vaccine, do we leave them to fend for themselves or have mandatory vaccination for children to go to schools, or for staff in care homes? There are many difficult questions. Transparency and regular reporting concerning safety and efficacity of vaccines under development may ensure the creation of trust and acceptance in vaccines.

Though a medically approved vaccine is not available yet, this is the time to formulate strategies for effective distribution and vaccine administration for each country. It is important to involve stakeholders from each country to ensure the individualized strategies are feasible. Vaccine acquisition, distribution, and uptake issues are substantially different in the developing world.

The WHO is working with the epidemic response group, Cepi, and the Vaccine Alliance of governments and organizations, known as Gavi, to try to level the playing field. At least 80 rich nations and economies, so far, have signed up for the global vaccine plan known as Covax, which aims to raise $2 billion by the end of 2020 to help buy and fairly distribute COVID-19 vaccines worldwide.

One important aspect for implementing COVID-19 vaccination programs in low-income countries is the cost. High cost of vaccines has limited HPV vaccination in Sub-Saharan countries [77]. We need affordable vaccines that are produced in sufficient quantity for use in every corner of the world.

## 10. Conclusions

The idea of a vaccine against SARS-CoV-2 in a fast-spreading pandemic has great allure. The sheer variety of the novel types of vaccines being investigated and the approaches they use, whatever form that takes, could serve us well for future outbreaks of other novel diseases.

At this point, it remains unclear how effective a COVID-19 vaccine will be, and if it will consist of a one-time vaccination or be seasonal, like the flu vaccine. Even if a promising vaccine surfaces by 2021 and can be mass-produced, the search will not end there. We are likely to need more than one vaccine to meet global demand.

Of importance, if SARS-CoV-2 remains a threat to a few, it will remain a threat to everybody. Politics and private interests should not hamper vaccine development and deployment efforts. This behemoth undertaking must be achieved through altruistic partnerships between industry, governments, and international organizations for the universal benefit of human health.

## Figures and Tables

**Table 1:** Vaccine development platforms against SARS-CoV-2, their viral targets, manufacturing description, advantages, and disadvantages.

(A) Vaccine Type	(B) Target	(C) Description	(D) Advantages	(E) Limitations
Live attenuated	Whole virus	Live viruses are weakened to reduce virulence, selected by growth in heterologous species and/or in tissue culture cells.	Induction of strong B and T cell responses	Risk for infection remains due to genetic reversion. They can cause disease in immune compromised individuals.
			Single administration without adjuvant is sufficient to induce protective immunity	Storage at cooler temps
			Often confers long-term immunity	
Inactivated whole virus	Whole virus	Whole virus is inactivated by chemical or physical procedures.	Risk of infectivity is eliminated without destroying antigenicity	Booster(s) may be required thus increasing cost
		Often administered with adjuvants.	Vaccine is stable.	Use of adjuvants may cause unwanted inflammatory response.
			Safe as no live virus is present.	
Subunit	S protein	Made of specific viral proteins or groups of proteins. Can be purified directly from viral particles.	Safe as viral particles cannot induce infection.	Induce insufficient cellular immunity.
			Viral proteins chosen likely to be immunogenic, inducing protecting antibodies,	Immune responses become weaker over time.
				Booster shots may be necessary thus increasing the cost of vaccination.
VLP-based (Subunit)	S protein	Viral surface antigens naturally occurring or synthesized are self-assembled into VLPs.	Present antigens in a dense, repetitive manner, enabling the cross-linking of B cell receptors.	Risk of presence of host cell-derived particles.
			Stimulate protective neutralizing antibodies.	Challenges to produce VLPs with optimal quality, stability, and good immunogenicity at high yield.
			Self-adjuvating properties.	
			Safe because VLPs do not induce infection.	
Vector-based	S protein	Gene encoding a major viral antigen is inserted (cloned) into another, non-virulent viral vector expressing introduced protein.	Long term gene expression.	Large-scale manufacturing of viral vectors may be expensive.
			Stimulates both humoral and cellular immune responses against introduced antigen.	Recombinant viruses may cause disease in immunocompromised hosts.
				Pre-existing antibodies to the vector may misdirect immune responses to vaccinating antigen.
DNA-based	S protein	Genetically engineered plasmids containing DNA for viral antigens.	Rapid production capacity.	Weaker induction of immunity.
		Relies on in situ production of the target antigen.	Induction of B and T cell responses.	Risk for integration into recipient’s chromosomal DNA resulting insertional mutagenesis.
			Long shelf life.	Require specific delivery devices which increases cost of administration.
			Heat stable as compared to RNA vaccine.	
			Inexpensive to produce.	
			No risk of infection.	
RNA-based	S protein	RNA sequence for viral antigens administered by carriers such as lipid nanoparticles.	Stimulates both cellular and humoral immunity.	Encoding only some fragments instead of whole virus limiting its immunogenicity.
			Direct delivery into the cytosol may enhance antigen expression.	Lack of interaction with endosomal RNA receptors may weaken immunostimulation.
			May be designed to be self-adjuvating.	Necessity to keep at cooler temperatures.
			Less likely to cause adverse effects such as allergies.	Vaccine delivery and uptake challenging in vivo.
			Does not interact with the genome.	
			Rapid production capacity.	

## Data Availability

Data sharing is not applicable to this article as no datasets were generated or analyzed during the current study.
